# Involvement of Th2 (interleukin-4) and Tumor necrosis factor-alpha in bowel disease pathogenesis through CD23/ no pathway: immunomodulatory effect of retinoic acid

**DOI:** 10.1186/2045-7022-4-S2-P60

**Published:** 2014-03-17

**Authors:** hayet Rafa, Mourad Belkhelfa, Houria Saoula, Yvan de Launoit, M’hamed Nakmouche, Nadira Delhem, Chafia Touil-Boukoffa

**Affiliations:** 1 Team: cytokines and No synthesis, university of sciences and Technology USTHB, BP 32 AL ALIA BAB EZZAOUAR FSB/LBCM/USTHB, Algiers, 16111, Algeria; 2 Team: cytokines and No synthesis, university of sciences and Technology USTHB, Laboratory of Cellular and Molecular Biology, Algiers, Algeria; 3 Maillot Hospital, department of Gastroenterology, Algiers, Algeria; 4 Université Lille-Nord de France, Institut de Biologie de Lille, UMR 8161, CNRS, Ins, Lille, France

## Background

Inflammatory bowel diseases (IBD) are chronic inflammatory diseases of the gastrointestinal tract, which clinically present as one of two disorders, Crohn's disease (CD) and ulcerative colitis (UC). The CD23 is a multifunctional molecule expressed on various cells. It is know as the low-affinity receptor for the Fc portion of IgE. It expression at the cell surface of phagocytic cells has been associated with the development of many inflammatory processes. The aim of this work was to study the involvement of Th2 (IL-4) and TNF-α in bowel disease pathogenesis through the CD23/NO Pathway in Algerian patients with IBD.

## Patients and methods

Twenty Patients with active UC, 15 patients with active CD and 10 healthy controls were enrolled in this study. We investigated in vivo IL-4 and TNF-α production in IBD patients. We also investigated the Th2 markers, TNF-α, CD23 and IgE using transcriptomic analysis. CD23 expression was also evaluated in inflamed colonic mucosa and PBMC using Immunohistochimistry and Western blot respectively. In the same way, we also studied in vitro the induction of nitric oxide production by Cross linking of CD23 by monoclonal antibody in the absence or presence of retinoic acid in cultures of Peripheral blood mononuclear cells (PBMC) and colonic mucosa from patients (CD and UC).

## Results

Our results indicate that IL-4/TNF-α production in IBD patients was increased in comparison to healthy donors. Transcriptomic Analysis of Th2 markers, TNF-α, CD23 and IgE revealed that levels of mRNA transcripts of the indicated genes are elevated in all IBD groups. A marked increase CD23 expression was seen in the inflamed colonic mucosa and PBMC from active UC and CD patients. In particular, CD23 expression markedly increased in samples from active UC patients. Our results show that cross linking of CD23 by monoclonal antibody up regulated NO production by PBMC and colonic mucosa. Retinoic acid down regulated the CD23 induced NO production ex vivo for the two groups of patients.

## Conclusion

These results suggest that Th2 (IL-4) and TNF-α play a pivotal role in IBD pathogenesis disease through CD23/nitric oxide pathway. Collectively our study suggests that Retinoic acid seem to be a useful tool for development of therapeutic strategies in IBD.

